# Routine Invasive vs Conservative Management of NSTEMI in Elderly Patients: Updated Meta-Analysis of Randomized Trials

**DOI:** 10.1016/j.jacadv.2025.102275

**Published:** 2025-10-29

**Authors:** Abiodun Idowu, Olayinka Adebolu, Endurance Evbayekha, Kevin Bryan Lo, Emmanuel Akintoye, Emmanuel Otabor, Michael Hamilton, Festus Ibe, Mohammad Al-Madani, Sahil Banka

**Affiliations:** a Department of Medicine, Jefferson Einstein Philadelphia Hospital, Philadelphia, Pennsylvania, USA; b Division of Internal Medicine, St. Luke’s Hospital, St. Louis, Missouri, USA; c Division of Cardiovascular Medicine, Brigham and Women’s Hospital Heart and Vascular Center, Boston, Massachusetts, USA; d Cardiovascular Medicine, Yale University School of Medicine, New Haven, Connecticut, USA; e Division of Interventional Cardiology, Jefferson Einstein Philadelphia Hospital, Philadelphia, Pennsylvania, USA

**Keywords:** coronary angiography, elderly, myocardial infarction, percutaneous coronary intervention

## Abstract

**Background:**

Decision-making on managing non–ST-segment elevation myocardial infarction (NSTEMI) with routine invasive strategy or conservatively in elderly patients remains a question of much debate, given that these patients are often under-represented in clinical trials.

**Objectives:**

In patients aged 70 years or more, is routine invasive strategy superior to conservative management of NSTEMI?

**Methods:**

We systematically reviewed multiple online databases to identify randomized controlled trials that evaluated the outcomes of invasive vs conservative strategies for NSTEMI in patients aged over 70 years. An inverse-variance weighting, frequentist meta-analysis was performed on data extracted from eligible studies.

**Results:**

A total of 8 randomized controlled trials involving 3,275 patients (49.7% invasive-treated and 50.3% conservative-treated) were included. Routine invasive strategy was not superior to conservative management in elderly patients with NSTEMI in all-cause mortality (OR: 1.07; 95% CI: 0.90-1.26; *P* = 0.44; I^2^: 0%) and cardiac-related death (OR: 1.05; 95% CI: 0.86-1.29; *P* = 0.64; I^2^: 0%). Routine invasive strategy, however, confers significant benefits in reducing the risk of myocardial reinfarction (OR: 0.71; 95% CI: 0.58-0.86; *P* = 0.0005; I^2^: 34%) and urgent revascularization (OR: 0.31; 95% CI: 0.23-0.42; *P* < 0.00001; I^2^: 0%) without predisposing NSTEMI patients aged ≥70 years to increased susceptibility to cerebrovascular accident (OR: 1.13; 95% CI: 0.82-1.55; *P* = 0.45; I^2^: 0%).

**Conclusions:**

In elderly patients with NSTEMI, routine invasive strategy does not improve primary outcomes of all-cause mortality or cardiac-related death but may reduce recurrent myocardial infarction and the need for urgent revascularization.

The prevalence of coronary artery disease increases with age, with elderly patients constituting a large proportion of patients presenting with non–ST-segment elevation myocardial infarction (NSTEMI).^1^ In 2024, 9.2% (32 million) of Americans were aged ≥70 years, and this proportion is predicted to continue increasing.^2^ Decision-making on managing NSTEMI invasively or conservatively in elderly patients is a question of much debate because this population is often under-represented in clinical trials. The few available trials are affected by slow enrollment and eventual premature termination, leading to being underpowered. These challenges have led to a lack of specific invasive management guidelines for elderly patients with NSTEMI. The European Society of Cardiology and American Heart Association/American College of Cardiology guidelines encouraged individualized care in older patients with NSTEMI based on patients’ preferences, risk assessment, and the presence or lack of high-risk features.^3^^,^^4^ This translates to a wide variation in the management strategies for elderly patients with NSTEMI, with some reluctance to utilize an invasive strategy due to unclear risk-to-benefit ratio.^5^

This study was therefore conducted to evaluate the outcomes of routine invasive vs conservative management for patients aged 70 years and over with NSTEMI. Routine invasive strategy refers to coronary angiography with the intent to revascularize with either percutaneous coronary intervention (PCI) or coronary artery bypass grafting (CABG) alongside standard medical therapy. The conservative strategy refers to optimal medical treatment without routine coronary angiography.

## Methods

### Ethics

This analysis conformed to the Code of Ethics of the World Medical Association for experiments involving human subjects. As a study-level meta-analysis that utilized data from publicly available published studies, Institutional Review Board approval and patient consent were not required for this study.

### Study design and protocol

This systematic review adhered to the Preferred Reporting Items for Systematic Reviews and Meta-Analyses guidelines.^6^ We prospectively registered the review in the International Prospective Register of Systematic Reviews under identification number CRD42025635084.

### Data sources and searches

We queried PubMed, EMBASE, Cochrane Library Central Register Controlled Trial, and ClinicalTrials.gov from inception to December 3, 2024, for randomized controlled trials (RCTs) that evaluated outcomes of invasive vs conservative strategies for NSTEMI in elderly patients. The searched keywords were (“elderly” OR “frail” OR “adults” OR “age >70”) AND (“NSTEMI” OR “non-ST-segment elevation myocardial infarction” OR “non-ST-elevation acute coronary syndrome”) AND (“coronary revascularization” OR “coronary intervention” OR “percutaneous coronary intervention” OR “conservative strategy” OR “invasive strategy”). No language or publication year filter was applied.

### Study selection

We downloaded eligible studies into Covidence (Veritas Health Innovation), a web-based collaboration software platform that streamlines the production of systematic and other literature reviews. Two authors (A.O. and I.A.) independently screened titles and abstracts of retrieved articles. The same 2 investigators then read the full texts of retained citations. A third reviewer (O.E.) resolved discrepancies. The bibliographies of eligible articles and previous similar systematic reviews were also examined to identify additional studies.

### Inclusion criteria

The prespecified inclusion criteria were as follows. 1) Population: individuals aged 70 years or more with NSTEMI. 2) Intervention: routine invasive strategy (diagnostic coronary angiography with revascularization as indicated). 3) Comparison: conservative strategy (standard medical therapy alone). Patients in conservative arm were allowed revascularization if they had refractory chest pain, life-threatening arrhythmia, hemodynamic instability, or worsening heart failure. 4) Outcomes: primary outcomes of interest were all-cause mortality and cardiac-related death. Secondary endpoints were myocardial reinfarction, urgent revascularization, stroke, and major bleeding at maximal available follow-up. 5) Study designs: RCTs that reported at least one outcome of interest.

### Data extraction and quality assessment

Two independent authors (I.A. and A.O.) extracted data using a predesigned Microsoft Excel sheet form. Data collected were study characteristics (author name, year of publication, country where the study was done, and sample size), patient’s baseline comorbidities (sex, prior myocardial infarction, prior CABG or PCI, hypertension, type 2 diabetes mellitus, hyperlipidemia, chronic kidney disease, atrial fibrillation, and current tobacco smoking), discharge medications, and outcomes of interest. We planned to assess publication bias with a funnel plot and Begg’s test if more than 10 studies were included.^7^ Risk of bias was evaluated as “low risk,” “unclear risk,” or “high risk” across the Revised Cochrane Risk-of-Bias Tool for Randomized Trials domains.^8^

### Statistical analysis

An inverse-variance weighting and random-effects model meta-analysis was performed for the included studies in line with the DerSimonian and Laird method in anticipation of heterogeneity in eligible studies.^9^ Heterogeneity was assessed by I^2^ statistics, with an I^2^ of <40% signifying no or minimal, and ≥40% representing moderate to large heterogeneity.^10^ Fixed-effect model analysis was applied when I^2^ is <40% (minimal heterogeneity). All statistical analyses were conducted at a 2-sided 5% significance level at 95% CI via Review Manager 5.4 (Cochrane Collaboration). As most of the included trials did not report time-to-event analysis of outcomes of interest, we pooled effect estimates by calculating OR from the reported frequency of events.

## Results

### Study characteristics

**Figure 1:**
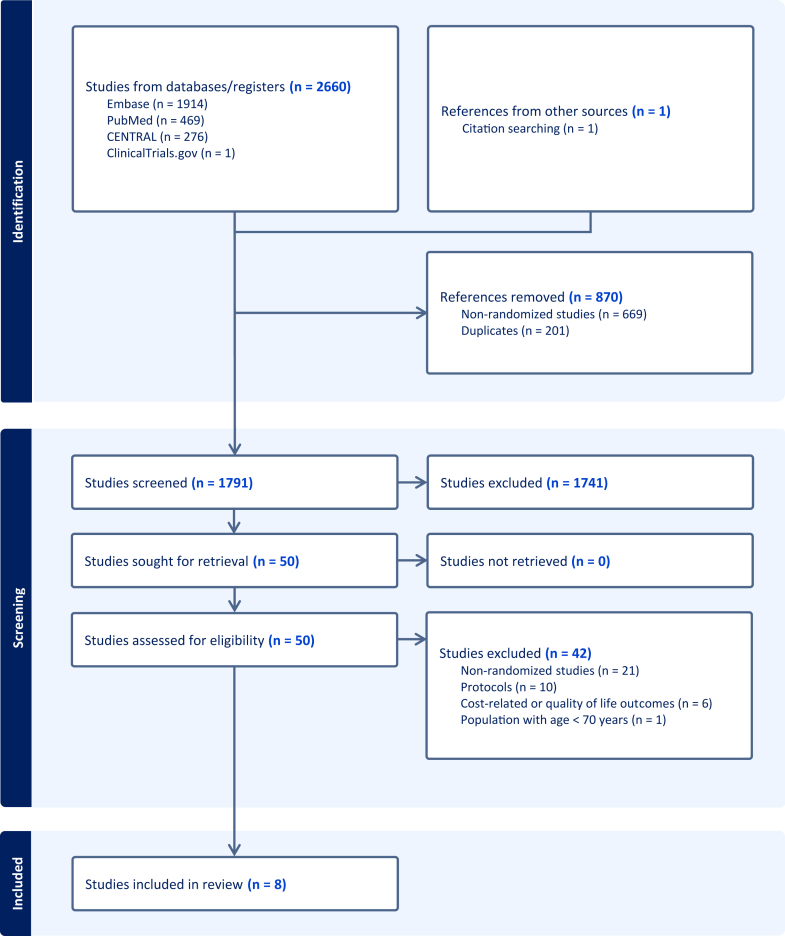
PRISMA Flowchart PRISMA = Preferred Reporting Items for Systematic Reviews and Meta-Analyses.

The search identified 2,661 studies. After the screening phase, 8 RCTs involving 3,275 patients (49.7% [n = 1,628] in the invasive and 50.3% [n = 1,647] in the conservative arm) were included in the analysis (Figure 1).^11^, ^12^, ^13^, ^14^, ^15^, ^16^, ^17^, ^18^ We did not conduct Begg’s test or funnel plot to evaluate the risk of publication bias as fewer than 10 studies met inclusion eligibility. All the studies were high-quality RCTs except Bach et al a subanalysis of TACTICS-TIMI 18 (Treat Angina with Aggrastat and determine Cost of Therapy with an Invasive or Conservative Strategy-Thrombosis In Myocardial Infarction 18 trial), that we could not evaluate its quality due to a lack of necessary information (Supplemental Table 1).

### Patient’s baseline characteristics

Prevalence of prior myocardial infarction ranged from 23% to 47%, and 11% to 40% had prior PCI. Hypertension was present in 57% to 94% of patients, diabetes mellitus in 14% to 60%, 2.2% to 9.2% were active tobacco smokers, and 10 to 31% had atrial fibrillation (Table 1). In the invasive group, 88% to 100% had coronary angiograms with 46.6% to 61.3% treated with PCI and CABG in 1 to 5.8%. In the conservative arm, 0 to 48.9% had cardiac catheterization with 0 to 22% treated with PCI and CABG in 0% to 11.5% (Supplemental Table 2). At discharge, 72% to 97% were prescribed aspirin, 65% to 96% with a P2Y12 receptor antagonist, 80% to 93% on statins, and 54% to 90% taking beta-blockers (Supplemental Table 3). Follow-up duration ranged from 6 months to 5.3 years.

**Table 1:** Baseline Characteristics of Participants in Included Studies

First Author, Year	n	Mean Age, y	Female	Prior MI	Prior CABG	Prior PCI	HTN	DM	AF	Smoking	CHF
Berg et al, 2023^11^											
Invasive	229	84.7	47.6%	46.7%	19.2%	24%	57.2%	19.7%	21.4%	7.9%	33.1%
Conservative	228	84.9	49.1%	39.5%	14%	20.2%	61%	14%	22.8%	9.2%	33.8%
Sanchis et al, 2024^12^											
Invasive	84	86 ± 5	62%	23%	6%	23%	92%	60%	31%	3.6%	15.5%
Conservative	83	85 ± 5	43%	39%	13%	40%	92%	52%	23%	2.4%	19.3%
Sanchis et al, 2016^13^											
Invasive	52	81 ± 5	44%	46%	19%	23%	94%	46%	27%	7.7%	15.5%
Conservative	54	83 ± 6	50%	43%	7%	17%	85%	46%	22%	3.7%	19.3%
Savonitto et al, 2012^14^											
Invasive	154	82 ± 4	51%	28%	11%	11%	92%	38%	15%	NA	10%
Conservative	159	82 ± 5	49%	34%	7.6%	20%	85%	41%	12%	NA	8.9%
Hirlekar et al, 2020^15^											
Invasive	93	84^a^	49.5%	31.9%	20.4%	16.3%	59.1%	17.2%	10.9%	2.2%	10.8%
Conservative	93	84^a^	40.9%	44.6%	15.1%	17.2%	63.4%	21.5%	18.5%	3.4%	8.6%
de Belder et al, 2021^16^											
Invasive	124	84.8	48.4%	26.8%	9.7%	17.2%	70.2%	26.6%	NA	8.1%	NA
Conservative	126	85.2	46%	28.5%	8.1%	12.9%	66.1%	15.2%	NA	3.3%	NA
Kunadian et al, 2024^18^											
Invasive	753	83 ± 5	44.8%	32.8%	13.4%	21.7%	65.1%	30.8%	13.5%	4.7%	9.7%
Conservative	765	82 ± 5	44.7%	29.7%	10.5%	18.2%	65.4%	30.6%	13%	6%	9.2%
Bach et al, 2004^17^											
Invasive	139	NA	NA	NA	NA	NA	NA	NA	NA	NA	NA
Conservative	139	NA	NA	NA	NA	NA	NA	NA	NA	NA	NA

AF = atrial fibrillation; CABG = coronary artery bypass graft; CHF = congestive heart failure; DM = diabetes mellitus; HTN = hypertension; MI = myocardial infarction; n = sample size; NA = not applicable; PCI = percutaneous coronary intervention.

a Median.

### All-cause mortality

**Figure 2:**
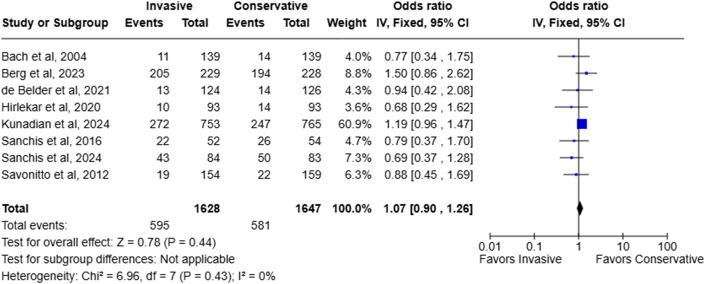
Forest Plot Comparing All-Cause Mortality Rate of Invasive Vs Conservative Management Strategy for NSTEMI in Patients Aged ≥70 Years NSTEMI = non–ST-segment elevation myocardial infarction.

All 8 RCTs reported the incidence of all-cause death with no significant between-study heterogeneity (I^2^: 0%). The mortality rate was 36.5% (595/1,628) in the invasive group compared to 35.3% (581/1,647) in the conservative group. The all-cause mortality rate was not significantly different between the 2 treatment strategies (OR: 1.07; 95% CI: 0.90-1.26; *P* = 0.44) (Figure 2).

### Cardiac-related death

**Figure 3:**
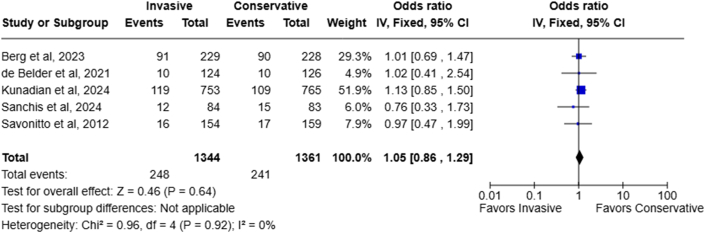
Forest Plot of Cardiac-Related Death in NSTEMI Elderly Patients Managed Invasively Vs Conservatively Abbreviation as in Figure 2.

Five RCTs reported cardiac-related death with no significant between-study heterogeneity (I^2^: 0%). The pooled effect estimate showed no significant difference in the rate of cardiac death for both management strategies (invasive 18.5% vs conservative 17.7%; OR: 1.05; 95% CI: 0.86-1.29; *P* = 0.64) (Figure 3).

### Myocardial reinfarction

**Figure 4:**
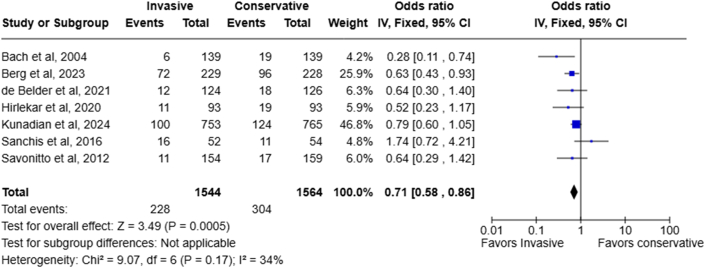
Forest Plots Showing the Odds Ratio of Myocardial Reinfarction

Seven RCTs provided data on the myocardial reinfarction rate with minimal heterogeneity (I^2^: 34%). Compared to the conservative strategy, the risk of myocardial reinfarction was significantly reduced by invasive treatment (14.8% in the invasive arm vs 19.4% in the conservative arm, OR: 0.71; 95% CI: 0.58-0.86; *P* = 0.0005) (Figure 4).

### Unplanned revascularization

**Figure 5:**
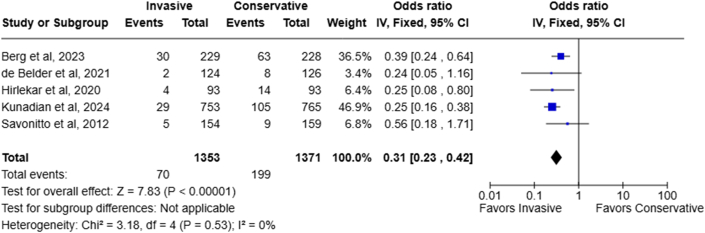
Forest Plot Showing Urgent Revascularization Rate for Patients Managed Via Invasive Strategy Vs Conservatively

Based on data from 5 RCTs with no between-study heterogeneity (I^2^:0%), the need for unplanned revascularization was significantly higher in the conservative treatment group. Unplanned revascularization occurred in 5.2% (70/1,353) of patients treated invasively vs 14.5% (199/1,371) managed conservatively (OR: 0.31; 95% CI: 0.23-0.42; *P* < 0.00001) (Figure 5).

### Cerebrovascular accident

**Figure 6:**
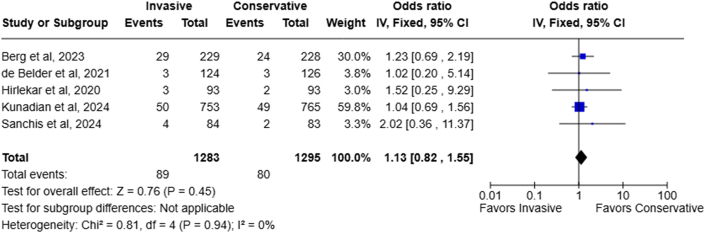
Forest Plot Showing the Odds Ratio of Stroke Incidence in Invasive Vs Conservative Treatment Groups

Five RCTs with no between-study heterogeneity (I^2^: 0%) reported stroke or transient ischemic attack rate after each treatment strategy. The 2 strategies had no significant difference in post-treatment stroke rate (6.9% in invasive treatment compared to 6.2% in conservatively treated patients, OR: 1.13; 95% CI: 0.82-1.55; *P* = 0.45) (Figure 6).

### Major bleeding complications

**Figure 7:**
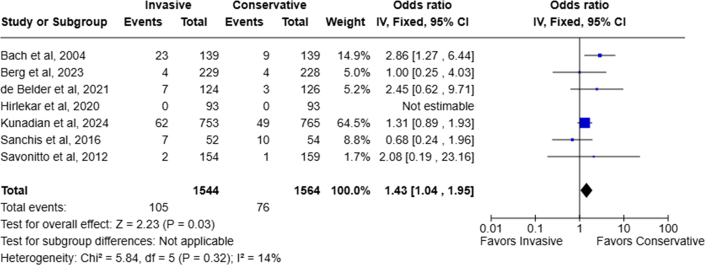
Major Bleeding Complication Rate of Invasive Vs Conservative Management Strategy for NSTEMI in Patients Aged ≥70 Years Abbreviation as in Figure 2.

Seven RCTs documented the number of patients with bleeding complications. Significantly more patients experienced major bleeding events in the routine invasive group, 6.8% (105 of 1,544 patients), compared to 4.9% (76 of 1,564 patients) in the conservative strategy arm (OR: 1.43; 95% CI: 1.04-1.95; *P* = 0.03; I^2^: 14%) (Figure 7). Applying a continuity correction of “0.5” for double zero-entry in event by Hirlekar et al, bleeding risk remains significantly higher in the routine invasive group (OR: 1.42; 95% CI: 1.04-1.94; *P* = 0.03).

### Sensitivity analysis of all outcomes based on the age of patients

Irrespective of the patient's age (≥70, ≥75, or ≥ 80 years); all-cause mortality, cardiac-related death, and stroke were not different between the 2 treatment strategies (*P* > 0.05) while the odds of myocardial reinfarction and need for unplanned revascularization was significantly lower in the routine invasive-treated groups compared to conservative arm (*P* < 0.05). On the other hand, major bleeding was significantly higher in those aged 70 to 79 years but had an insignificant trend in patients aged ≥80 years (Supplemental Table 4).

## Discussion

This meta-analysis found that compared to conservative management (optimal medical therapy alone) of NSTEMI in patients aged ≥70 years, routine invasive strategy was not superior for primary outcomes of all-cause mortality or cardiac-related death. Invasive strategy, however, may reduce the risk of myocardial reinfarction and urgent revascularization. Both treatment strategies had similar cerebrovascular accident complication rates, but major bleeding was significantly higher among patients treated with routine invasive strategy compared to those treated with conservative treatment (Central Illustration).

**Central Illustration:**
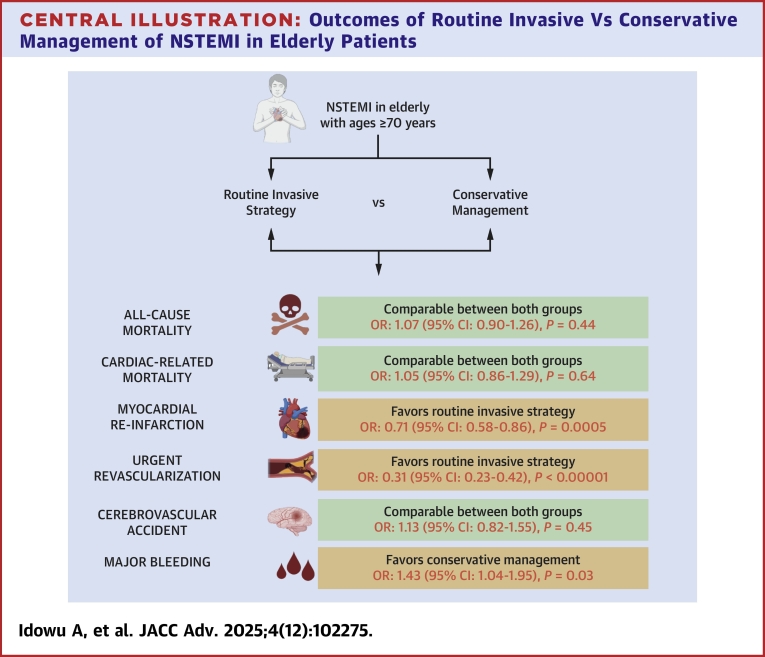
Outcomes of Routine Invasive Vs Conservative Management of NSTEMI in Elderly Patients Abbreviation as in Figure 2.

We found no statistically significant difference in all-cause and cardiac-related mortality between those managed invasively and those treated conservatively across ages over 70, 75, and 80 years. The absence of mortality benefits for the routinely invasively treated group compared to conservative management in this study contrasts with prior multiple observational data,^19^, ^20^, ^21^, ^22^ and some meta-analyses.^23^^,^^24^ Unlike our study, these meta-analyses pooled results from more observational studies than RCTs. We hypothesized that their results were likely driven by observational studies, which, by design, have confounding bias and are prone to selection bias. This hypothesis is supported by a meta-analysis by Hu et al, where a subgroup analysis showed no mortality difference based on 5 RCTs, while pooled results from 22 observational studies indicated reduced mortality in the invasively treated group.^25^ Our study's lack of significant difference in mortality outcomes between the 2 management strategies provides evidence for physicians and patients to make informed, evidence-based decisions. From a mortality perspective, conservative management may be as effective as routine invasive strategies in patients aged over 70 years with NSTEMI. A plausible reason for the neutral mortality outcome with the routine invasive strategy in this study is likely related to the timing of intervention, as only a few trials had angiography within 24 hours (Supplemental Table 2) and complete revascularization rate among patients who underwent coronary interventions is low as shown by Kunadian et al trial where 55.9% had multivessel disease but only 29.9% and 3.3% had multivessel PCI and CABG, respectively.^18^ In the FIRE (Functional Assessment in Elderly MI Patients with Multivessel Disease) trial, physiology-guided complete revascularization was associated with a significant reduction in mortality compared to culprit-vessel-only revascularization in patients over 75 years.^26^

However, from the standpoint of reducing the risk of myocardial reinfarction and the need for urgent revascularization, a routine invasive strategy was found to be superior to optimal medical therapy alone (conservative management). This is consistent with previous registry data^27^^,^^28^ and smaller sample meta-analyses.^29^^,^^30^ Similarly, 3 (Berg et al,^11^ Hirlekar et al,^15^ and Kunadian et al^18^) of the 4 RCTs with results on urgent revascularizations showed a reduced risk of unplanned revascularization in routine invasive-treated patients. The only RCT (de Belder et al^16^), with equitable results for both treatment strategies, had a low event rate partly because it was terminated early due to a slow recruitment rate.^16^ This finding implies some clinical benefits of using an invasive strategy over conservative management in elderly patients with NSTEMI.

Similar to prior meta-analyses, we found that the rate of cerebrovascular accidents across the spectrum of ages ≥70 to 79 years and ≥80 years was identical after either treatment strategy. Unlike those previous meta-analyses, this updated analysis demonstrated a significantly increased rate of major bleeding complications in routine invasive-treated patients. The RCTs, except one, reported no bleeding difference in both groups. The study by Bach et al^17^ more than 2 decades ago, was the only study that reported significantly higher bleeding in the routine invasive group. A recent trial, including those containing objectively 32.4% frail and 62% cognitively impaired participants, failed to demonstrate a higher bleeding tendency in the invasive group.^18^ This may partly be because, unlike older RCTs, recent trials predominantly utilized radial access, drug-eluting stents, and better antiplatelet therapies.

### Strengths and limitations

The results of our analysis should be interpreted with some limitations in mind. First, we used intention-to-treat analysis, although some patients (3.2% to 23%) in the conservative group crossed over to undergo revascularization. Additionally, not all patients in the invasive group had cardiac catheterization, and even fewer underwent revascularization (Supplemental Table 2). Since this is a study-level meta-analysis without access to individual patient data, we could not perform per-protocol or as-treated subanalyses to assess the impact of these crossovers on the outcomes of interest. Furthermore, while this study focused on hard endpoints like mortality, other outcomes like quality of life (not assessed in this study) are equally important for elderly patients. Another limitation was the lack of blinding in the RCTs due to the invasive nature of the interventions. Despite these limitations, a key strength of this updated meta-analysis is its exclusive focus on RCTs, which helps avoid selection and confounding biases common in non-RCTs. Additionally, including data from studies with patients exhibiting high levels of geriatric syndromes such as frailty, multimorbidity, and cognitive impairment strengthens the robustness of the findings, as these conditions are crucial in tailoring care for the elderly. Moreover, the inclusion of recent trials allows evaluation of outcomes based on current therapies and advances in interventional practices.

## Conclusions

Our study showed that the routine invasive approach is not superior to the conservative strategy in terms of mortality outcomes in elderly patients aged ≥70 years with NSTEMI. However, it may reduce the risk of myocardial reinfarction and the need for urgent revascularization without predisposing to an increased risk of cerebrovascular accident. This information is useful for risk-benefit discussions to help decide on an optimal personalized approach to NSTEMI treatment in an elderly patient.

Perspectives **COMPETENCY IN SYSTEMS-BASED PRACTICE:** These findings support current guideline recommendations for individualized decision-making in elderly NSTEMI patients, especially given the heterogeneity in physiologic reserve in this population.

**COMPETENCY IN INTERPERSONAL AND COMMUNICATION SKILLS:** Effective shared decision-making with elderly patients and their caregivers is essential to align treatment strategies with personal goals, functional status, and quality-of-life expectations.

**TRANSLATIONAL OUTLOOK:** Future research should explore whether complete revascularization strategies improve survival in elderly NSTEMI patients.

## Funding support and author disclosures

The authors have reported that they have no relationships relevant to the contents of this paper to disclose.
