# Supplementary material for: Routine Invasive vs Conservative Management of NSTEMI in Elderly Patients: Updated Meta-Analysis of Randomized Trials
Source: JACC Adv. 2025 Oct 29;4(12):102275. doi: 10.1016/j.jacadv.2025.102275 (PMC12805171; doi:10.1016/j.jacadv.2025.102275)
Supplement: Supplemental Tables 1-4 [file mmc1.docx]

**Supplemental Appendix**

|  | **Berg et al, 2023^11^** | **Sanchis et al, 2024**  ^12^ | **Sanchis et al, 2016**  ^13^ | **Savonitto et al, 2012**  ^14^ | **Hirlekar et al, 2020**  ^15^ | **de Belder et al, 2021**  ^16^ | **Kunadian et al, 2024**  ^18^ | **Bach et al, 2004**  ^17^ |
| --- | --- | --- | --- | --- | --- | --- | --- | --- |
| Bias arising from the randomization process | 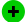 | 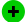 | 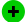 | 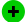 | 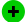 | 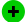 | 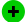 | 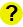 |
| Bias due to deviations from intended interventions | 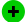 | 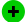 | 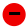 | 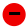 | 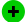 | 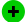 | 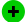 | 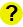 |
| Bias due to missing outcome data | 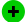 | 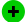 | 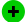 | 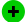 | 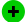 | 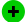 | 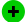 | 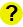 |
| Bias in measurement of the outcome | 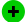 | 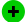 | 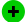 | 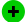 | 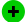 | 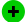 | 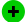 | 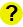 |
| Bias in selection of the reported result | 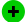 | 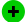 | 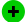 | 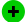 | 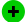 | 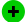 | 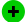 | 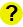 |


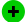
 - Low risk of bias,
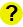
 - Unclear risk of bias,
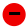
 - High risk of bias

**Supplemental Table 1**: Quality assessment of included RCTs using the RoB2 tool

| **Studies** | **Treatment strategy** | **Sample size** | **CAG** | **PCI** | **CABG** | **Timing of CAG from admission** |
| --- | --- | --- | --- | --- | --- | --- |
| Berg et al, 2023 ^11^ | Invasive | 229 | 96.1% | 46.7% | 2.6% | Within 24 hours |
|  | Conservative | 228 | 0 | 0 | 0 |  |
| Sanchis et al, 2024 ^12^ | Invasive | 84 | 97.6% | 57.1% | 2.4% | within 72 hours |
|  | Conservative | 83 | 10.8% | 9.6% | 0 |  |
| Sanchis et al, 2016 ^13^ | Invasive | 52 | 100% | 53.8% | 3.8% | Within 72 hours |
|  | Conservative | 54 | 20% | 7.4% | 1.9% |  |
| Savonitto et al, 2012 ^14^ | Invasive | 154 | 88.3% | 49.4% | 5.8% | within 72 hours |
|  | Conservative | 159 | 28.9% | 22% | 0.6% |  |
| Hirlekar et al, 2020 ^15^ | Invasive | 93 | 95.7% | 61.3% | 1% | Not specified |
|  | Conservative | 93 | 4.3% | 3.2% | 1.1% |  |
| de Belder et al, 2021 ^16^ | Invasive | 124 | 92.7% | 49.2% | 1.6% | Not specified |
|  | Conservative | 126 | 8.7% | 2.4% | 0.8% |  |
| Kunadian et al, 2024 ^18^ | Invasive | 753 | 90.3% | 46.6% | 3.3% | within 3 to 7 days |
|  | Conservative | 765 | none | none | none |  |
| Bach et al, 2004 ^17^ | Invasive | 139 | 95% | 42.5% | 19.4% | Within 48 hours |
|  | Conservative | 139 | 48.9% | 20.9% | 11.5% |  |

**Supplemental Table 2**: Coronary angiogram and revascularization data in the included studies

*Legends:* n – sample size; CAG – coronary angiography; PCI – Percutaneous coronary intervention; CABG – Coronary artery bypass graft surgery

| **Author, year** | **Strategy** | **n** | **ASA** | **P2Y12i** | **DOAC** | **Warfarin** | **Statin** | **Beta-blocker** | **ACEi/ARB** |
| --- | --- | --- | --- | --- | --- | --- | --- | --- | --- |
| Berg et al, 2023 ^11^ | Invasive | 229 | 94.8% | 72.5% | 1.7% | 21.8% | 91.3% | 85.6% | 52.4% |
|  | Conservative | 228 | 93% | 72.8% | 3.9% | 14% | 84.2% | 84.6% | 53.9% |
| Sanchis et al, 2024 ^12^ | Invasive | 84 | 94.8% | 69% | 23% | 9.5% | 89.3% | 66.7% | 56% |
|  | Conservative | 83 | 84.3% | 65% | 23% | 13.3% | 89.2% | 78.3% | 61.4% |
| Sanchis et al, 2016 ^13^ | Invasive | 49 | 74% | 65% | NA | NA | 82% | 54% | 80% |
|  | Conservative | 51 | 72% | 77% | NA | NA | 93% | 68% | 74% |
| Savonitto et al, 2012 ^14^ | Invasive | 154 | 92% | 73% | NA | NA | NA | NA | NA |
|  | Conservative | 159 | 97% | 86% | NA | NA | NA | NA | NA |
| Hirlekar et al, 2020 ^15^ | Invasive | 93 | 91% | 88.9% | NA | 10.3% | 79.8% | 87.8% | 64% |
|  | Conservative | 93 | 89% | 88% | NA | 16.7% | 81.3% | 90.2% | 72.8% |
| de Belder et al, 2021 ^16^ | Invasive | 124 | 95.9% | 95.9% | 3.3% | 5.8% | 80.3% | 86.9% | 72.9% |
|  | Conservative | 126 | 88.5% | 95.1% | 6.6% | 12.3% | 70.5% | 82.5% | 70.5% |
| Kunadian et al, 2024 ^18^ | Invasive | 753 | 90.7% | 89.6% | 15% | 3.7% | 90.2% | 79.2% | 71.2% |
|  | Conservative | 765 | 87% | 94.4% | 17% | 4.5% | 90% | 78.6% | 78.6% |
| Bach et al, 2004 ^17^ | Invasive | NA | NA | NA | NA | NA | NA | NA | NA |
|  | Conservative | NA | NA | NA | NA | NA | NA | NA | NA |

**Supplemental Table 3:** Discharge medications

*Legends*: n- sample size, ASA- aspirin, P2Y12i- P2Y12 receptor inhibitors, DOAC - direct oral anticoagulant, ACEi/ARB- angiotensin-converting enzyme inhibitors/ angiotensin receptor blockers, NA – No available data

| **Outcomes** | **Age** | **OR** | **95% CI** | **p-value** | **I^2^ test** |
| --- | --- | --- | --- | --- | --- |
| All-cause mortality | > 70 years | 1.07 | 0.90 – 1.26 | 0.44 | 0% |
|  | > 75 years | 1.12 | 0.94 – 1.34 | 0.19 | 0% |
|  | > 80 years | 1.12 | 0.75 – 1.68 | 0.59 | 20% |
| Cardiac death | > 70 years | 1.05 | 0.86 – 1.29 | 0.64 | 0% |
|  | > 75 years | 1.07 | 0.87 – 1.32 | 0.52 | 0% |
|  | > 80 years | 1.01 | 0.72 – 1.43 | 0.95 | 0% |
| Myocardial re-infarction | > 70 years | 0.71 | 0.58 – 0.86 | **0.0005** | 34% |
|  | > 75 years | 0.67 | 0.55 – 0.82 | **0.0001** | 0% |
|  | > 80 years | 0.61 | 0.45 – 0.84 | **0.003** | 0% |
| Urgent Revascularization | > 70 years | 0.31 | 0.23 – 0.42 | **0.00001** | 0% |
|  | > 75 years | 0.31 | 0.23 – 0.42 | **0.00001** | 0% |
|  | > 80 years | 0.36 | 0.23 – 0.55 | **0.00001** | 0% |
| Stroke | > 70 years | 1.13 | 0.82 – 1.55 | 0.45 | 0% |
|  | > 75 years | 1.11 | 0.80 – 1.53 | 0.53 | 0% |
|  | > 80 years | 1.23 | 0.73 – 2.07 | 0.44 | 0% |
| Major bleeding | > 70 years | 1.43 | 1.04 – 1.96 | **0.03** | 14% |
|  | > 75 years | 1.53 | 1.11 – 2.13 | **0.01** | 0% |
|  | > 80 years | 1.57 | 0.59 – 4.20 | 0.36 | 0% |

**Supplemental Table 4:** Sensitivity analysis of outcomes based on the age of patients (Invasive strategy was the experimental arm while the conservative group was the control)
